# Long-Term Asymptomatic Medial Migration of a Tympanostomy Tube into the Middle Ear: A Case Report and Review of the Literature

**DOI:** 10.3390/reports9040343

**Published:** 2026-10-11

**Authors:** Nikolaos Fylaktou, Athanasios Vlachodimitropoulos, Gerasimos Danielides, Georgios Batsaouras, Spyridon Lygeros

**Affiliations:** Department of Otorhinolaryngology, School of Health Sciences, University of Patras, 26504 Rion, Greece

**Keywords:** tympanostomy tube, grommet, medial migration, middle ear, case report

## Abstract

**Background and Clinical Significance:** Tympanostomy tubes (TTs) are normally extruded into the external auditory canal within 6–18 months, whereas medial migration into the middle ear is a rare complication. Medialized tubes have been associated with otalgia, otorrhea, conductive hearing loss, granuloma and perilymphatic fistula, yet may remain clinically silent; no consensus exists on whether asymptomatic medialized tubes should be removed. **Case Presentation:** A 24-year-old man attended a routine otorhinolaryngological examination without otological complaints. He had undergone bilateral TT insertion at four years of age for otitis media with effusion, with complete resolution and no further otological procedures. Otoscopy demonstrated an intact tympanic membrane through which a round, blue structure was visible within the posteroinferior quadrant of the tympanic cavity; otoendoscopy confirmed a foreign body in contact with the middle ear mucosa. Tympanometry showed a type A curve and pure-tone audiometry showed normal, symmetrical thresholds. High-resolution computed tomography showed an aerated middle ear cleft, well pneumatized mastoid cavities, an intact ossicular chain and no radiological features of chronic otitis media. Conservative management was adopted, with clinical and audiological review planned at 6–12-month intervals; at the most recent review, three months after diagnosis, the patient remained asymptomatic. **Conclusions:** A medially migrated TT may be retained within the middle ear for two decades without inflammation, structural damage or hearing loss. In selected patients with an intact tympanic membrane, normal audiometry and unremarkable imaging, structured observation is a defensible alternative to removal, provided the patient is counseled about the symptoms that should prompt review.

## 1. Introduction and Clinical Significance

Tympanostomy tube (TT) insertion is one of the most frequently performed procedures in otorhinolaryngology, with millions of insertions carried out worldwide each year [1,2]. It is used principally in the management of otitis media with effusion (OME), recurrent acute otitis media (AOM) and chronic Eustachian tube dysfunction, providing immediate restoration of middle ear ventilation and improvement in hearing [1,2]. Although the technique is generally considered safe and well tolerated, it carries a range of potential complications, most of which are mild and self-limiting, such as otorrhea, transient postoperative pain or prolonged tube retention beyond the expected duration [1,2]. Current clinical practice guidelines provide detailed indications, contraindications and follow-up recommendations for TT placement in children [3].

Less common but clinically significant complications include persistent tympanic membrane perforation, cholesteatoma formation and alterations in tube position [1,2]. Extrusion of the tube into the external auditory canal is a common and expected event that represents the normal end of the life cycle of the tube. By contrast, medial displacement of the tube into the middle ear—internationally referred to as medial migration—is an extremely rare and undesirable complication, with only limited reports available in the literature [1,2]. It describes movement of the tube from the surface of the tympanic membrane into the tympanic cavity, typically occurring after insertion without immediate recognition of the complication [4,5,6,7]. Despite its rarity, medial migration may remain completely asymptomatic or may lead to a wide spectrum of clinical manifestations, ranging from chronic otorrhea to conductive hearing loss. Diagnosis is often incidental, made during routine otoscopic examination or during imaging performed for unrelated reasons [8,9,10].

Management strategies reported in the international literature vary considerably and depend on symptomatology, the exact location of the tube within the middle ear and the perceived risk of long-term complications. Whereas some authors advocate surgical removal in order to prevent chronic inflammation or cholesteatoma formation [4,5,6,9,11], others support a conservative approach with close clinical follow-up in selected cases [8,11,12,13].

Within this context, the present report describes a young adult in whom a TT was identified within the middle ear as a result of medial migration of unknown timing following insertion in childhood, remaining undiagnosed and asymptomatic for approximately twenty years thereafter.

Clinical Significance: To the best of our knowledge, this case is distinguished not just by the duration of retention, which cannot be established since the exact timing of migration is unknown, but by the combination of complete absence of symptoms, normal audiometry and unremarkable imaging after an interval of approximately two decades since insertion, accompanied by an entirely benign clinical course. The case demonstrates that medial migration does not follow a uniform trajectory, that it may escape detection for long intervals because of mild or entirely absent symptoms, and that prolonged presence of the tube is not invariably accompanied by inflammatory or structural sequelae.

## 2. Case Presentation

A 24-year-old man presented for a routine otorhinolaryngological examination without any otological symptoms. His medical history revealed bilateral TT insertion at the age of four for the treatment of OME, with complete clinical and audiological resolution and no history of tube reinsertion or other otological procedures. During the intervening years, he reported no episodes of otalgia, otorrhea, aural fullness, tinnitus, vertigo, or subjective or objective hearing loss. He had no history of head trauma, barotrauma, diving or systemic disease.

**Figure 1:**
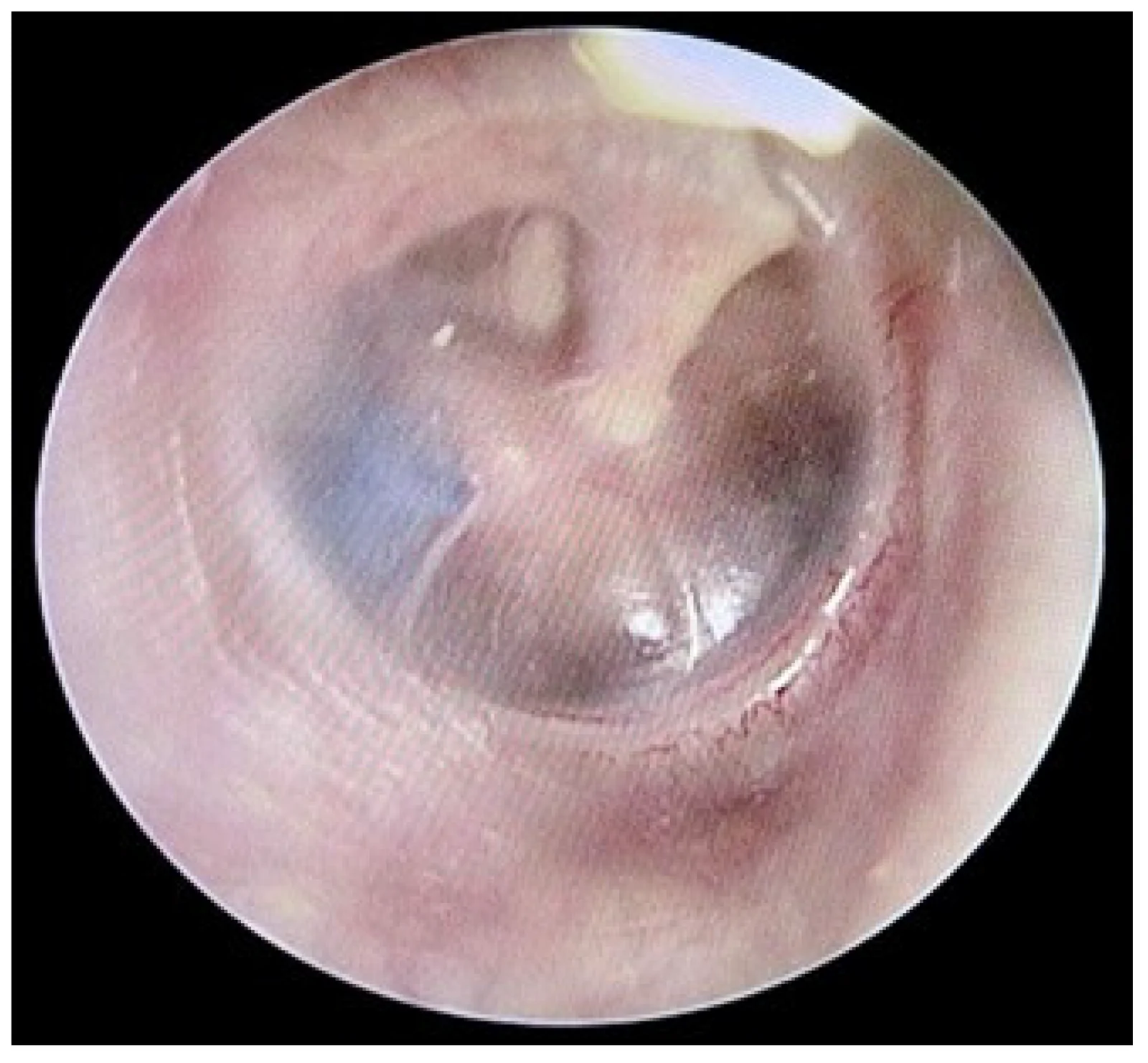
Otoendoscopic view of the right ear. The tympanic membrane is intact and of normal color, with no retraction, atrophy or myringosclerosis. A round, blue structure corresponding to the medially migrated tympanostomy tube is visible through the membrane within the posteroinferior quadrant of the tympanic cavity, in contact with the middle ear mucosa.

Otoscopic examination of the right ear demonstrated a normal auricle and external auditory canal. The tympanic membrane exhibited normal color and anatomy, without scarring, atrophy, retraction or signs of active pathology. However, a round, blue structure was observed within the middle ear cavity at the posteroinferior quadrant, visible through the intact tympanic membrane. Contralateral ear examination was unremarkable. This finding was consistent with medial migration of a TT. No mobility of the structure and no surrounding inflammatory change were observed. Otoendoscopic examination confirmed the presence of a foreign body within the tympanic cavity, in contact with the middle ear mucosa (Figure 1).

**Figure 2:**
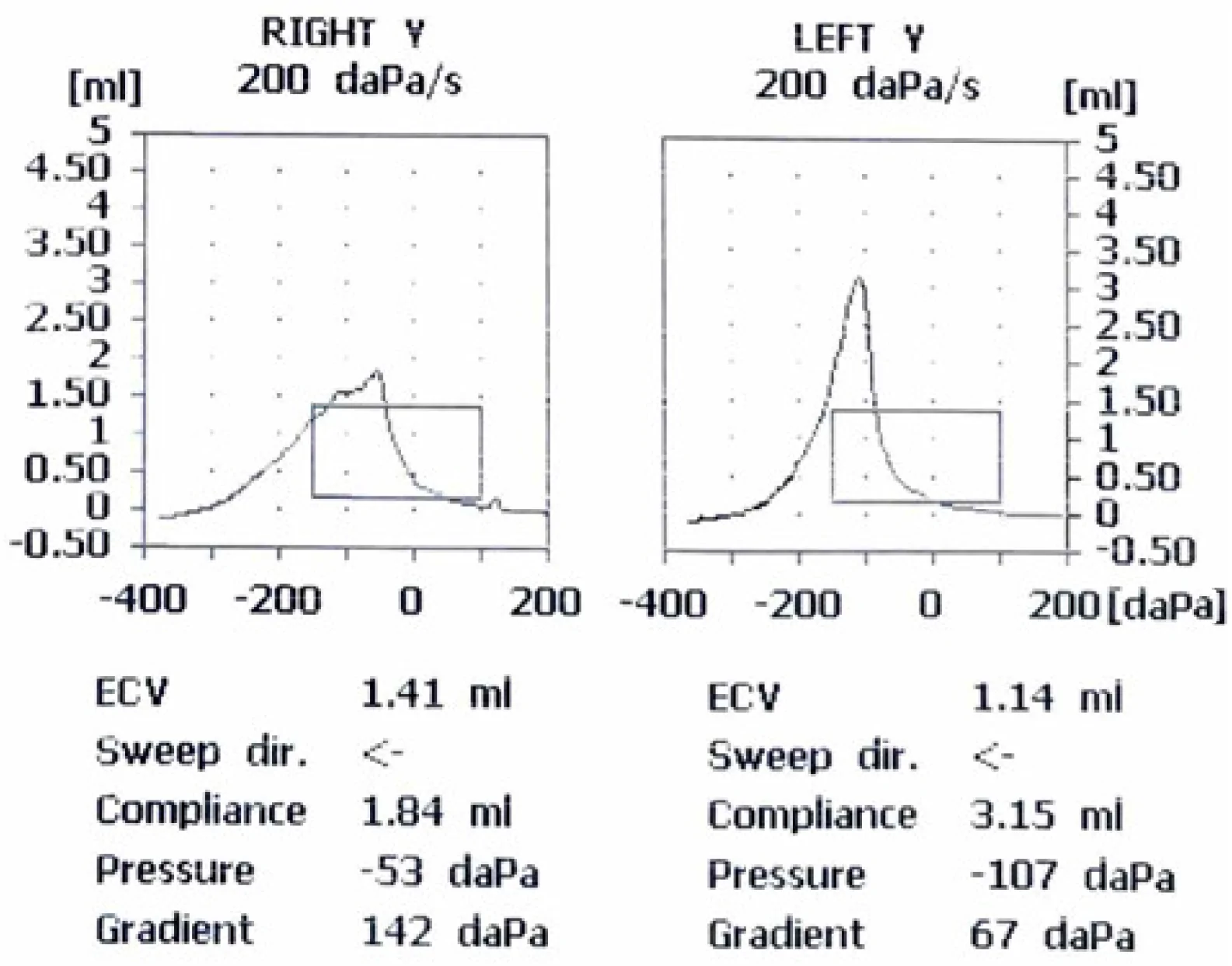
Conventional tympanometry. A type A tympanogram with normal compliance was obtained in the affected ear, with no evidence of middle ear effusion or Eustachian tube dysfunction.

**Figure 3:**
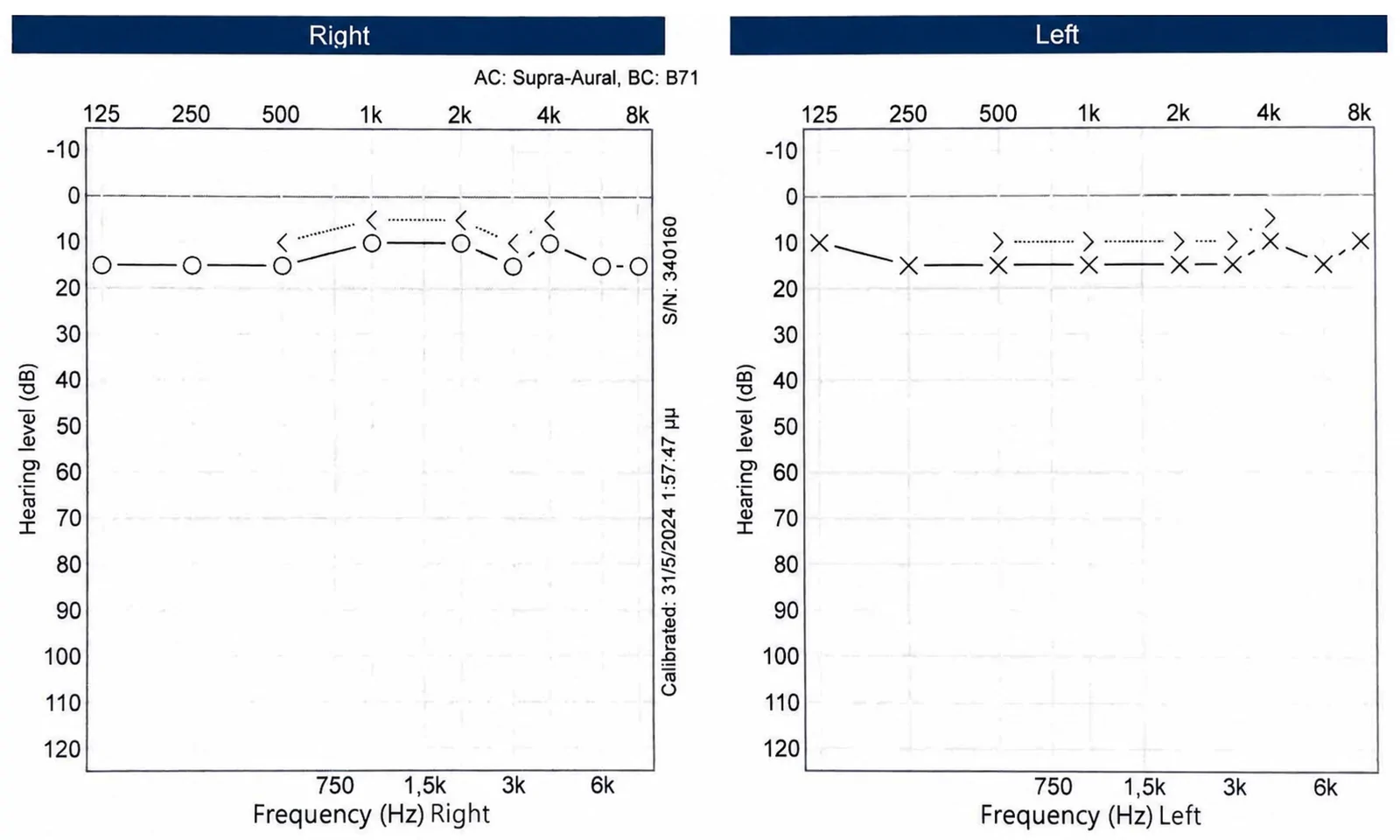
Pure-tone audiometry. Air- and bone-conduction thresholds are within normal limits and symmetrical across all tested frequencies (125–8000 Hz), with no air–bone gap and therefore no evidence of conductive hearing loss.

Conventional tympanometry revealed a type A tympanogram in both ears (peak pressure within −100 to +50 daPa); static compliance was within the device’s published adult reference range. Peak compliance and tympanometric width showed mild inter-aural asymmetry (right: 1.84 mL, 142 daPa; left: 3.15 mL, 67 daPa), which remained within this normal range bilaterally and is consistent with normal middle ear mechanics rather than a mass-loading or stiffening effect from the retained tube (Figure 2). Pure-tone audiometry demonstrated normal hearing thresholds bilaterally, with no air–bone gap and symmetrical hearing across all tested frequencies (Figure 3).

**Figure 4:**
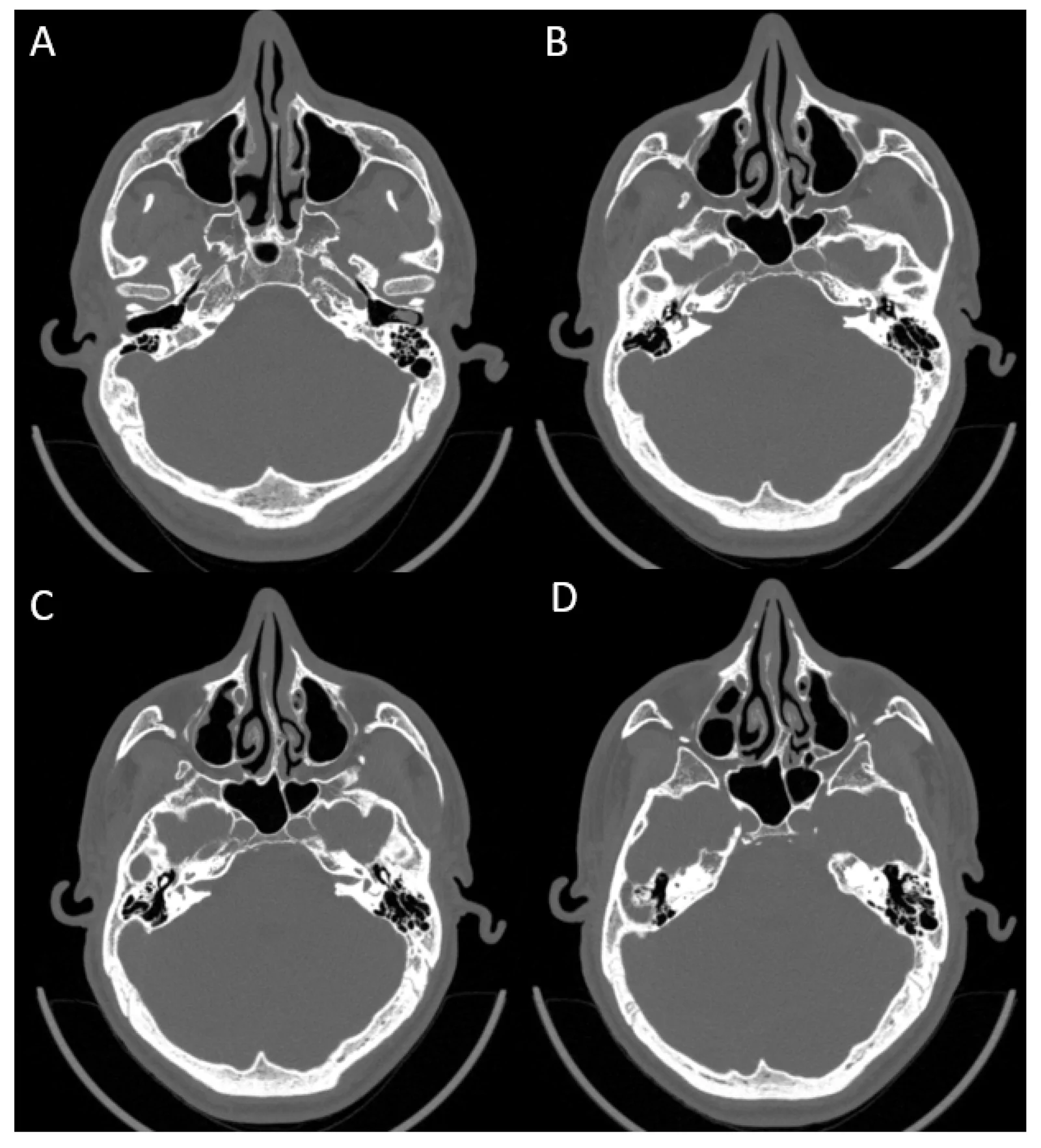
(**A**–**D**) Axial high-resolution computed tomography (HRCT) of the temporal bones at four representative levels, ordered from inferior to superior. The middle ear cleft is aerated and the mastoid air cells are well-pneumatized bilaterally, with an intact ossicular chain, no soft-tissue opacification, no scutum blunting or bony erosion, and no radiological features of chronic otitis media or cholesteatoma. The tympanostomy tube itself was not clearly resolved on CT; its presence and position were established by otoscopy and otoendoscopy.

For further evaluation, high-resolution computed tomography (CT) of the temporal bones was performed. The tube itself was not clearly resolved as a discrete radiopaque structure on CT, consistent with its small size and non-metallic composition; its presence and position had already been established otoscopically and endoscopically. CT nonetheless demonstrated a normal appearance of the middle ear cleft, without signs of inflammation, bony erosion or ossicular chain damage (Figure 4).

Given the complete absence of symptoms, normal hearing, absence of any inflammatory process and stability of the finding, a conservative management strategy with regular follow-up was adopted after a detailed discussion with the patient. Clinical and audiological reassessment every 6–12 months was recommended, together with counseling regarding potential future symptoms that would warrant prompt re-evaluation, including otalgia, otorrhea, tinnitus, hearing deterioration or vertigo. The option of surgical removal by tympanotomy was also discussed and would be offered in the event of symptom development or of a change in the position of the foreign body. At the most recent review, the patient remained entirely asymptomatic, with an intact tympanic membrane, a type A tympanogram and unchanged audiometric thresholds. The chronology of the episode of care is summarized in Table 1.

## 3. Discussion

TT insertion is one of the most commonly performed surgical procedures in otorhinolaryngology and is widely used for long-term ventilation of the middle ear cavity, particularly in children with persistent OME or recurrent AOM [1,2]. The normal course involves spontaneous extrusion into the external auditory canal, typically within 6–18 months, depending on the type and material of the tube. Most complications are mild and include otorrhea, myringosclerosis and persistent tympanic membrane perforation, whereas less common complications include granulomatous reactions, iatrogenic adhesions and cholesteatoma formation [1,2]. Medial migration of a TT represents one of the rarest complications of the procedure, with a reported incidence of the order of 0.5% of insertions or lower [2]. This pathological migration differs fundamentally from the expected external extrusion and is associated with mechanical or inflammatory factors that alter the integrity or the mechanical resistance of the tympanic membrane.

### 3.1. Review of Previously Reported Cases

To place the present case in context, a review of previously reported cases of medial migration of TTs was performed. PubMed/MEDLINE, Scopus, the Cochrane Library and Google Scholar were searched from inception to March 2026 using combinations of the terms “tympanostomy tube”, “grommet”, “ventilation tube”, “medial migration”, “medial displacement”, “foreign body” and “middle ear”. Reports were eligible for inclusion if they were published in English or French and provided extractable individual patient data, including age, sex, indication for tube insertion, interval to diagnosis, presenting features, management and outcome. Clinical and management data from eligible reports were extracted independently by two authors. Because the eligible literature consisted exclusively of case reports and small case series with heterogeneous, non-standardized outcome reporting, a formal systematic review following the Preferred Reporting Items for Systematic Reviews and Meta-Analyses (PRISMA) reporting guidance, with a risk-of-bias assessment, was not considered appropriate for this body of evidence; a structured narrative review was performed instead to synthesize the available reports and situate the present case within them.

A prior structured review by Bezdjian et al. [12] searched the English- and French-language literature to February 2014 and identified 10 studies reporting 29 patients. The present review extends this search to March 2026 and an additional database (Google Scholar). The demographic characteristics, clinical presentation, management strategies, and outcomes of the previously reported cases are summarized in Table 2.

The reported cases demonstrate considerable variability in clinical presentation, associated sequelae and management strategy. Most cases involve pediatric patients, consistent with the population in which TTs are most frequently inserted, although delayed diagnosis in adolescence or adulthood is well documented, often as an incidental finding during routine otoscopic examination or imaging. Reported clinical manifestations range from completely asymptomatic presentations to a variety of otological complications. Symptomatic cases most commonly presented with otorrhea, otalgia or conductive hearing loss [4,13,17]. Inflammatory sequelae described in the literature include COM, middle ear effusion and granulomatous foreign body reactions within the tympanic cavity [10,17,20]. More severe complications have also been reported, including ossicular chain involvement and perilymphatic fistula, likely due to local inflammation and mechanical pressure exerted by the migrated tube [9,10]. In addition, cholesteatoma formation has been described in association with TTs in general, raising the theoretical concern that a medially migrated tube could act as a nidus for squamous epithelial proliferation [2].

### 3.2. Proposed Pathogenesis

The exact pathogenesis of medial migration of TTs has not been fully elucidated. Proposed contributing factors include improper insertion technique, excessively deep or medial placement, an unfavorable myringotomy site, local inflammation or scar tissue formation, the presence of biofilms, and anatomical or developmental characteristics of the pediatric middle ear [5,6,7]. Attempted manipulation or instrumentation of a retained tube and negative middle ear pressure have also been implicated [4,5]. The present patient clearly differs from most published reports, as no inflammatory reaction, scarring or audiological impairment was observed despite the prolonged presence of the foreign body, which suggests that a tube retained within a well-pneumatized, non-inflamed middle ear may be tolerated indefinitely.

### 3.3. Diagnostic Evaluation

Otoscopy represents the first step in diagnosis [4,5], and visualization of a foreign body behind an intact tympanic membrane is the typical finding. Endoscopic examination provides improved illumination and magnification and facilitates assessment of associated abnormalities. Tympanometry and pure-tone audiometry are essential to document middle ear function and to exclude a conductive component. High-resolution CT of the temporal bone may aid in assessing the location of a migrated tube and its relationship to middle-ear structures when the tube is radiologically visible, while also allowing evaluation for complications such as cholesteatoma, ossicular involvement or bony erosion [4,10].

### 3.4. Management: Removal Versus Observation

The management of medial TT migration remains a subject of considerable debate. Some authors advocate surgical removal even in asymptomatic patients [4,5,6,9,11], aiming to prevent complications such as cholesteatoma, granulomatous hyperplasia, ossicular damage or fistula formation. This approach is based on the premise that long-term retention of a foreign body within the middle ear is potentially hazardous and that removal by tympanotomy—increasingly performed endoscopically [15]—generally carries a low risk. In contrast, several authors support a conservative follow-up strategy when the patient is asymptomatic, the tympanic membrane is intact, hearing is normal and imaging findings are unremarkable [8,11,12,13]. This approach is based on the observation that in many cases the presence of the tube does not provoke inflammatory or anatomical change, whereas surgical intervention introduces a small but real risk of tympanic membrane perforation, ossicular injury or sensorineural hearing loss, in addition to the risks of general anesthesia in children. A survey of pediatric otolaryngologists confirmed that practice is divided and that the decision is largely driven by symptoms and by the position of the tube [11].

**Figure 5:**
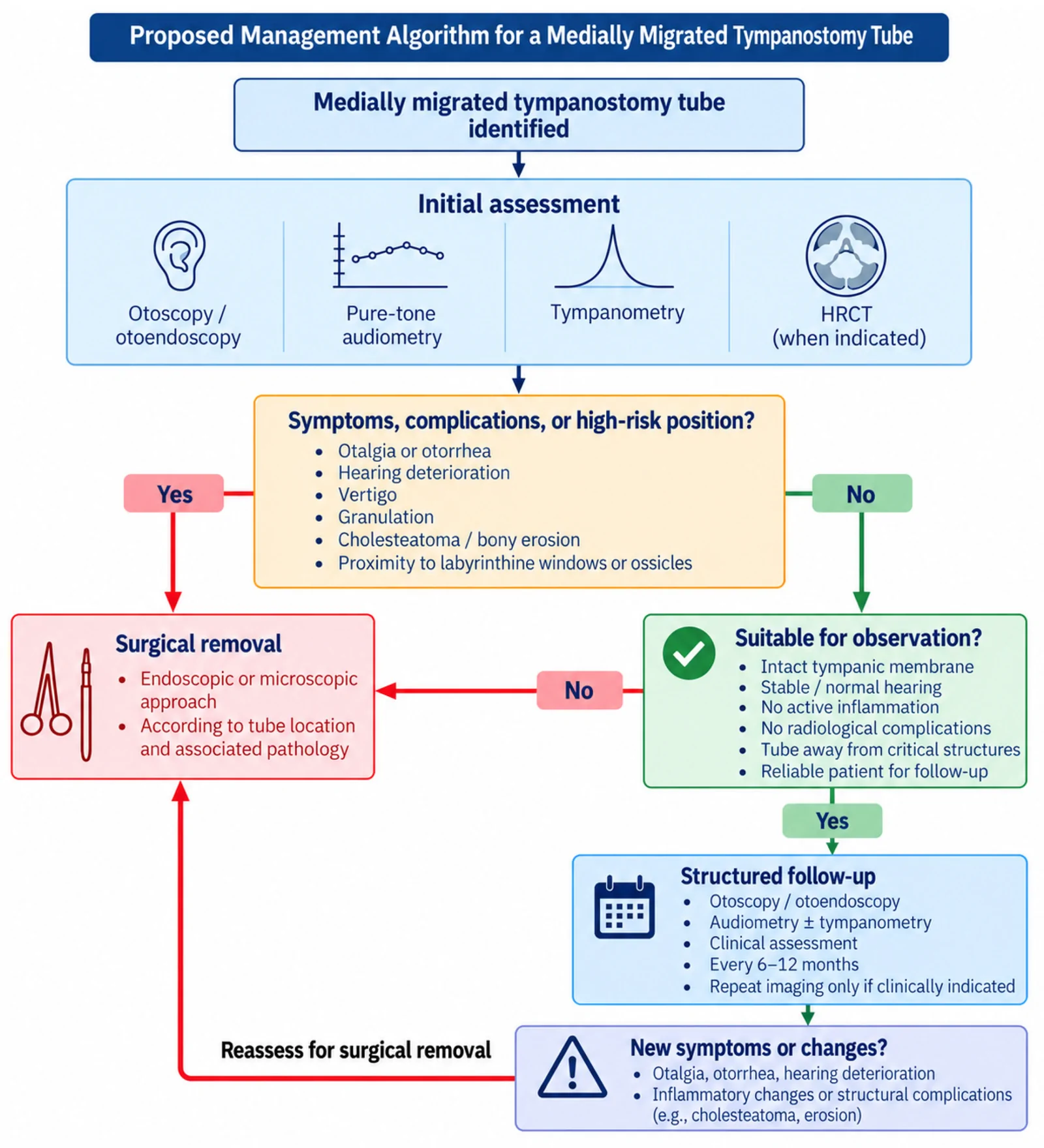
Proposed management algorithm for a medially migrated tympanostomy tube. The algorithm summarizes a pragmatic approach based on the available literature and the present case, integrating clinical, audiological and radiological findings into the decision between surgical removal and structured observation.

Drawing on this synthesis, the literature and the present case point to a set of factors clinicians may weigh when a medially migrated TT is identified. Features favoring removal include otological symptoms (otalgia, otorrhea, aural fullness, vertigo or subjective hearing loss), a measurable air–bone gap, imaging evidence of ossicular involvement, bony erosion, granulation tissue or cholesteatoma, and a tube that is mobile, partially extruded, or impinging on the ossicular chain or a labyrinthine window. Features favoring continued observation include a completely asymptomatic patient, an intact tympanic membrane without inflammatory change, a normal tympanogram with symmetrical audiometric thresholds, imaging showing a well-aerated, non-inflamed middle ear cleft with no ossicular or bony abnormality, and a tube that remains stable and non-mobile on serial examination. In this latter scenario, we suggest clinical and audiological reassessment every 6–12 months, imaging only if new findings or symptoms emerge, and explicit counseling of the patient about symptoms that should prompt re-evaluation and reconsideration of removal. A proposed practical management algorithm incorporating these clinical, audiological and radiological considerations is presented in Figure 5. The proposed algorithm is intended as a pragmatic aid to shared decision-making rather than a validated evidence-based guideline, given the limited and heterogeneous nature of the available literature.

### 3.5. Limitations

This report describes a single patient and therefore cannot support generalized recommendations. The exact date of migration could not be established, and the interval of approximately twenty years is inferred from the age at insertion. Retrospective symptom reporting over such a long interval is subject to recall bias, and no imaging from the intervening period was available to document the stability of the tube position over time. The accompanying literature review is narrative rather than systematic; most published reports are isolated cases, reporting is heterogeneous and incomplete, and publication bias probably favors symptomatic or complicated cases, which may make the true course of medial migration appear more severe than it is. Finally, the limited available literature makes it difficult to establish clear management guidelines [11,12]. Future multicentre registries or retrospective studies may help to clarify risk factors and contribute to the development of a standardized management strategy.

## 4. Conclusions

Medial migration of a TT into the middle ear is a rare but clinically significant complication that may be recognized many years after the initial insertion. The present case demonstrates that the clinical course of this complication is not uniform and that, in selected patients, a TT retained within the middle ear may remain completely asymptomatic, without inflammatory or anatomical change and without any impact on hearing.

Long-term retention without complication supports the view that conservative management with careful follow-up may be a safe option in patients with an intact tympanic membrane, normal audiological assessment and unremarkable imaging. The continuing controversy in the literature regarding surgical versus non-surgical management emphasizes the need for an individualized approach based on the clinical and audiological characteristics of each patient, on the position of the tube, and on informed patient preference. More systematic documentation of such cases, ideally through multicentre registries, is required to identify risk factors and to develop clearer management algorithms.

## Figures and Tables

**Table 1:** Timeline of the episode of care. HRCT, high-resolution computed tomography; OME, otitis media with effusion; PTA, pure-tone audiometry; TM, tympanic membrane; TT, tympanostomy tube.

Time Point	Events, Findings and Interventions
Age 4 years	Bilateral TT insertion for OME; uneventful postoperative course, with complete clinical and audiological resolution of the effusion.
Age 4–24 years (≈20 years)	No otalgia, otorrhea, aural fullness, tinnitus, vertigo or hearing loss. No tube reinsertion and no further otological procedure or consultation. Neither extrusion of the tube nor its retention was documented at any point.
Age 24 years, index visit	Routine otorhinolaryngological examination in an asymptomatic patient. Otoscopy: intact TM of normal color and anatomy with a round, blue structure in the posteroinferior quadrant of the tympanic cavity. Otoendoscopy: foreign body in contact with the middle ear mucosa (Figure 1).
Index visit (same day)	Tympanometry: type A curve with normal compliance (Figure 2). PTA: normal, symmetrical thresholds with no air–bone gap (Figure 3).
1 week after index visit	HRCT of the temporal bones: well-pneumatized middle ear cleft, no inflammation, bony erosion, ossicular involvement or cholesteatoma (Figure 4).
1 week after index visit	Shared decision-making: conservative management adopted; 6–12-monthly clinical and audiological review scheduled; counseling on symptoms warranting prompt re-evaluation; tympanotomy offered should symptoms or tube position change.
Most recent review (3 months)	Entirely asymptomatic; intact TM, type A tympanogram and unchanged audiometric thresholds.

**Table 2:** Demographic data, clinical presentation, management and outcome of previously reported cases of medial migration of tympanostomy tubes, together with the present case.

Study	Sex/Age (y)/Side	Indication; Tube Type	Interval (mo)	Presenting Features	Management	Outcome
Ahluwalia et al. [14]	NA/NA/unilat.	ETD; NA	NA	Asymptomatic	Removal	NA
	NA/NA/unilat.	ETD; NA	NA	Asymptomatic	Observation	NA
Wongwan et al. [10]	F/37/R	OME; NA	24	Otorrhea, profound SNHL, aural polyp, granuloma with intracranial extension	Removal + mastoidectomy	NA
Mukerji [5]	M/2/R	OME; Armstrong	1	Otalgia, type B tympanogram, mild CHL	Removal + reinsertion	2 y—asymptomatic
Benchafai et al. [6]	M/10/L	NA; Shepard	12	Aural fullness, mild CHL	Removal	NA
Malic et al. [15]	M/5/R	NA; NA	16	Asymptomatic	Endoscopic removal	NA
Rinaldi et al. [16]	M/82/R	OME; NA	108	SNHL	Observation	12 mo—asymptomatic
Shakeel et al. [17]	F/40/R	OME; T-tube	96	Otalgia, mucopurulent discharge, moderate CHL	Removal + reinsertion	NA
Yaman et al. [18]	NA/NA/unilat.	OME; Shepard	24	Asymptomatic	Observation	NA
	NA/NA/unilat.	OME; Shepard	24	Middle ear effusion	Removal	NA
Hajiioannou et al. [9]	M/65/R	OME; Shah	25	Aural fullness, HL, dizziness, PLF, bone erosion	Removal	1.25 mo—HL unchanged
Hamid [19]	M/45/L	NA; NA	180	Dizziness, BPPV	Observation	12 mo—asymptomatic
Abbarah and Abbarah [13]	F/32/R	OME; Richards	48	Plugged ear, muffled hearing, mild CHL, effusion	Removal + reinsertion	Asymptomatic, no recurrence
	F/18/R	OME; Richards	48	Plugged ear, effusion, tympanosclerosis, TM retraction, mild CHL	Removal + reinsertion	0.75 mo—asymptomatic
Groblewski and Harley [4]	M/3/R	RAOM; Armstrong	0.75	Asymptomatic	Removal + reinsertion	24 mo—tube extruded
	M/3/bilat.	RAOM; Donaldson	16	Speech delay; type B tympanogram (R)	Removal	16 mo—asymptomatic
	M/5/R	NA; Pope	NA	Speech delay, effusion, mild CHL	Removal	NA
	F/19/L	COM; Reuter Bobbin	120	Otalgia, otorrhea, dull TM, mild CHL	Removal + mastoidectomy	22 mo—retracted TM, granulation
	M/5/L	RAOM; Paparella type I	32	Speech delay	Removal	3 mo—asymptomatic
	M/7/R	COM; Armstrong	14	Asymptomatic	Removal	1 mo—asymptomatic
Roy et al. [20]	M/8/L	OME; NA	NA	Thickened TM, mild CHL	Removal	4 mo—asymptomatic
	M/6/R	OME; NA	NA	Type C tympanogram, CHL, atelectasis	Removal + reinsertion	12 mo—asymptomatic
	F/28/R	COM; NA	264	Mild CHL, effusion, granulation polyp	Removal	8 mo—improved
	M/3/R	OME; NA	16	Perforated TM, effusion, SNHL	Removal + paper patch	3 mo—asymptomatic
Kumar et al. [7]	M/6/R	OME; Shah	30	NA	Removal	NA
	M/11/R	COM; Shah	84	Perforated TM	Removal	NA
	F/48/L	COM; Shepard	12	Perforated TM	Removal	NA
Green et al. [8]	F/12/L	OME; NA	48	Asymptomatic	Observation	24 mo—asymptomatic
	M/12/R	RAOM; NA	12	Asymptomatic	Observation	30 mo—asymptomatic
	F/42/L	COM; NA	1.5/12/8	Middle ear effusion, CHL	Removal + reinsertion	8 mo—tube extruded
Cunningham et al. [21]	F/9/R	OME; NA	24	NA	Removal	NA
Present case	M/24/R	OME; NA	240	Asymptomatic; incidental finding	Observation	3 mo—asymptomatic, normal hearing

F, female; M, male; bilat., bilateral; mo, months; y, years; AOM, acute otitis media; BPPV, benign paroxysmal positional vertigo; CHL, conductive hearing loss; COM, chronic otitis media; ETD, Eustachian tube dysfunction; HL, hearing loss; L, left; NA, not available; OME, otitis media with effusion; PLF, perilymphatic fistula; R, right; RAOM, recurrent acute otitis media; SNHL, sensorineural hearing loss; TM, tympanic membrane; unilat., unilateral (side not stated in the source report). Where several values are separated by slashes in the interval column, the patient underwent more than one tube insertion and the interval is given for each.

## Data Availability

The original contributions presented in this study are included in the article. Further inquiries, including access to the underlying clinical and imaging data, can be directed to the corresponding author; these data are not publicly available in order to protect patient privacy.

## Abbreviations

The following abbreviations are used in this manuscript:

TT	Tympanostomy tube
OME	Otitis media with effusion
AOM	Acute otitis media
CT	Computed tomography
PRISMA	Preferred Reporting Items for Systematic Reviews and Meta-Analyses
COM	Chronic otitis media
HRCT	High-resolution computed tomography
PTA	Pure-tone audiometry
TM	Tympanic membrane
ETD	Eustachian tube dysfunction
SNHL	Sensorineural hearing loss
CHL	Conductive hearing loss
HL	Hearing loss
PLF	Perilymphatic fistula
BPPV	Benign paroxysmal positional vertigo
RAOM	Recurrent acute otitis media
